# Twelve Diseases That Changed Our World

**DOI:** 10.3201/eid1405.080072

**Published:** 2008-05

**Authors:** John W. Ward

**Affiliations:** * Centers for Disease Control and Prevention, Atlanta, Georgia, USA

**Keywords:** infectious diseases, public health, epidemics, book review

Twelve Diseases That Changed Our World offers engaging observations on a dozen diseases to serve 2 goals. The opening chapters meet the title’s promise by tracing the impact of hereditary blood disorders porphyria and hemophilia on the succession of European monarchs in the 16th through 18th centuries. Also presented is a riveting account of the consequences of a potato blight in 1840s Ireland, which forced migration of millions to England and North America. Thereafter, the book turns to the topic of infectious diseases and the lessons learned from earlier responses to “unanticipated outbreaks of disease” to inform preparedness for future outbreaks. Specifically, the chapters are devoted to the study of cholera, smallpox, bubonic plaque, syphilis, tuberculosis, malaria, fever, influenza, and AIDS. These topics are familiar territory for Dr. Sherman, who recently authored The Power of Plagues, in which he also examines 7 of these infections; his command of the subject matter is evident.

Each chapter is packed with information ranging from pathogenesis and clinical manifestations to epidemiologic calculations and antimicrobial drug resistance. A limited number of references are provided in the concluding book notes, grouped by chapter and page number, which offer additional resources for readers seeking more information. Of particular interest is the book’s accounting of 19th-century pioneers in epidemiology and infectious diseases. John Snow’s use of early epidemiologic tools to associate cholera deaths with water from the Broad Street pump, Louis Pasteur’s development of vaccines, and Robert Koch’s discovery of tubercle bacillus and the cholera vibro all get their deserved attention; Florence Nightingale’s use of numerical data to demonstrate improvements in patient hygiene comes as a pleasant surprise. A concise volume written for the general reader, Twelve Diseases That Changed Our World provides an excellent foundation for the study of public health and infection control.

